# CLN8 disease caused by large genomic deletions

**DOI:** 10.1002/mgg3.263

**Published:** 2016-11-23

**Authors:** Clare Beesley, Rita J. Guerreiro, Jose T. Bras, Ruth E. Williams, Ana Lia Taratuto, Christin Eltze, Sara E. Mole

**Affiliations:** ^1^ Regional Genetics LaboratoryGreat Ormond Street HospitalLondonWC1N 3BHUK; ^2^ Department of Molecular NeuroscienceInstitute of NeurologyUniversity College LondonQueen SquareLondonWC1N 3BGUK; ^3^ Department of Medical Sciences and Institute of Biomedicine ‐ iBiMEDUniversity of AveiroAveiro3810‐193Portugal; ^4^ Children's NeurosciencesEvelina London Children's HospitalWestminster Bridge RoadLondonSE1 7EHUK; ^5^ Institute for Neurological ResearchMontañeses 2325Buenos AiresArgentina; ^6^ Neurology DepartmentGreat Ormond Street HospitalGreat Ormond StreetLondonWC1N 3JHUK; ^7^ MRC Laboratory for Molecular Cell BiologyGenetics and Genomics Medicine UnitDepartment of Genetics, Evolution and EnvironmentInstitute of Child HealthUniversity College LondonGower StreetLondonWC1E 6BTUK

**Keywords:** Batten, CLN8, NCL, neuronal ceroid lipofuscinosis

## Abstract

**Background:**

The presence of deletions can complicate genetic diagnosis of autosomal recessive disease.

**Method:**

The DNA of patients was analyzed in a diagnostic setting.

**Results:**

We present three unrelated patients each carrying deletions that encompass the 37 kb *CLN8* gene and discuss their phenotype. Two of the cases were hemizygous for a mutant allele – their deletions unmasked a mutation in *CLN8* on the other chromosome.

**Conclusion:**

Microarray analysis is recommended in any patient suspected of NCL who is apparently homozygous for a mutation that is not present in one of the parents or when the family has no known consanguinity.

## Introduction

The neuronal ceroid lipofuscinoses are a group of progressive neurodegenerative disorders that mainly, but not exclusively, affect children. Mutations in 13 genes have been reported, with some cases still remaining genetically undiagnosed. Most are inherited as autosomal recessive. The type of mutation affects the age of onset of disease as well as its severity. A gene‐based nomenclature is increasingly being used (Williams and Mole 2012). Over 440 mutations have been reported in the NCLs and these are collected in the freely accessible NCL mutation database (www.ucl.ac.uk/ncl/).

Some NCL genes carry mutations that are shared across a population or even across continents, but many mutations are unique to one or a few families. In our research and diagnostic work that has screened over 900 patients for mutations in NCL genes over the last 10 years, we noticed several cases carried large genomic deletions that encompassed the *CLN8* gene (OMIM *607837), which we report here. The presence of deletions can complicate genetic diagnosis. One Irish patient has already been reported with a de novo terminal deletion of the short arm of chromosome 8p23.3 (Allen et al. 2012). One Turkish family carries a 2.6 kb intragenic deletion in *CLN8* that causes more severe disease than most other mutations (Reinhardt et al. 2010). To date, ~30 other mutations that cause CLN8 disease have been reported, of which 21 are missense mutations, one is a nonsense mutation, and five are small deletions of 1–3 bp. CLN8 disease is seen in many countries.

CLN8 belongs to the TLC superfamily that shares a 200 amino acid domain (amino acids 62–262) encompassing five transmembrane helices (Winter and Ponting 2002). Mutations that affect this domain tend to result in a severe phenotype typical of variant late infantile NCL presumably due to complete loss of function and even protein location (Kousi et al. 2012), although the missense mutation p.(Gln194Arg) is present in a patient with a slightly more severe disease course (Cannelli et al. 2006), and affected siblings with p.(Gln256Glu) have a noticeably varying disease course (Zelnik et al. 2007). Missense mutations that lie outside this domain, such as p.(Arg24Gly) can result in a much less severe phenotype such as Progressive Epilepsy with Mental Retardation (EPMR or Northern Epilepsy), first described in Finland (Hirvasniemi et al. 1994; Ranta et al. 1999). Most other missense mutations cause similar disease, although p.(Trp263Cys) is associated with a later age of onset and more protracted disease course (Kousi et al. 2012).

Here, we present three unrelated patients each carrying deletions that encompass the 37 kb *CLN8* gene, and discuss their phenotype. Two of the cases were hemizygous for a mutant allele – their deletions unmasked a mutation in *CLN8* on the other chromosome. We now recommend including microarray analysis in any patient suspected of NCL who is apparently homozygous for a mutation that is not present in one of the parents or when the family has no known consanguinity.

## Material and Methods

### Identification of subjects

One family was part of a research‐directed approach to identify the underlying disease genes in families diagnosed with NCL and contained within the DNA database at UCL. Blood from two further families suspected of NCL were received by the Regional Genetics Laboratory at Great Ormond Street Hospital for diagnostic testing.

#### Ethical compliance

The participation of all families was possible by their informed consent and all procedures employed were reviewed and approved by the appropriate institutional review committee.

### Illumina SNP beadchip analysis

To assess the presence of large structural variants or large regions of homozygosity (>1 Mb) in family A, the DNA sample was run on HumanOmniExpress BeadChips as per the manufacturer's instructions (Illumina Inc). Data were visualized and analyzed using the GenomeStudio Data Analysis Software (Illumina Inc., San Diego, CA, USA).

### DNA and mutation analysis

Genomic DNA was obtained from all subjects using standard procedures. All of the coding exons and intron/exon boundaries of the *CLN8* gene (NM_001042432.1) were amplified by PCR (primer sequences available on request) using Megamix (Microzone, Haywards Heath, UK). PCR products were purified with AmpureXP (Beckman Coulter, High Wycombe, UK) and then Sanger sequenced using BigDye Terminator v.3.1 technology (Life Technologies, Paisley, UK). Mutation Surveyor software (SoftGenetics, State College, PA, USA) was used to align traces to the reference sequence and call the variants/mutations. Alamut software (Interactive Biosoftware, Rouen, France) was used to investigate the potential pathogenicity of the variants by in silico software tools, for example, PolyPhen, SIFT, SNPs. Genome‐wide array CGH analysis for Family B was carried out using the NimbleGen 135k CGH microarray (Roche NimbleGen, Madison, WI, USA), scanned using GenePix Pro 7 (Molecular Devices, Sunnyvale, CA, USA), and processed using Infoquant Fusion software (Infoquant, London, UK).

## Results

### Child A (UCL303 Pa)

This family is from Argentina with no known consanguinity. The proband was 5 years old at time of blood collection, with one elder sibling who died at the age of 13 years with the same disease, one elder sibling who was unaffected, and a younger sibling whose disease status was unknown. She experienced seizures from 3 years and 8 months; ataxia from 4 years; myoclonus from 3 years 6 months; drop attacks from 4 years and neuro‐regression from 4 years of age; The visual evoked potential (VEP) was abnormal at 5 years; somatosensory evoked potential (SSEP) was said to be normal at 5 years. No further clinical details are available. Electron microscopy showed curvilinear inclusions (CL) in skin biopsy. Brain imaging showed cortical and cerebellar atrophy. These investigations confirmed a diagnosis of NCL but the underlying gene was not known.

**Figure 1:**
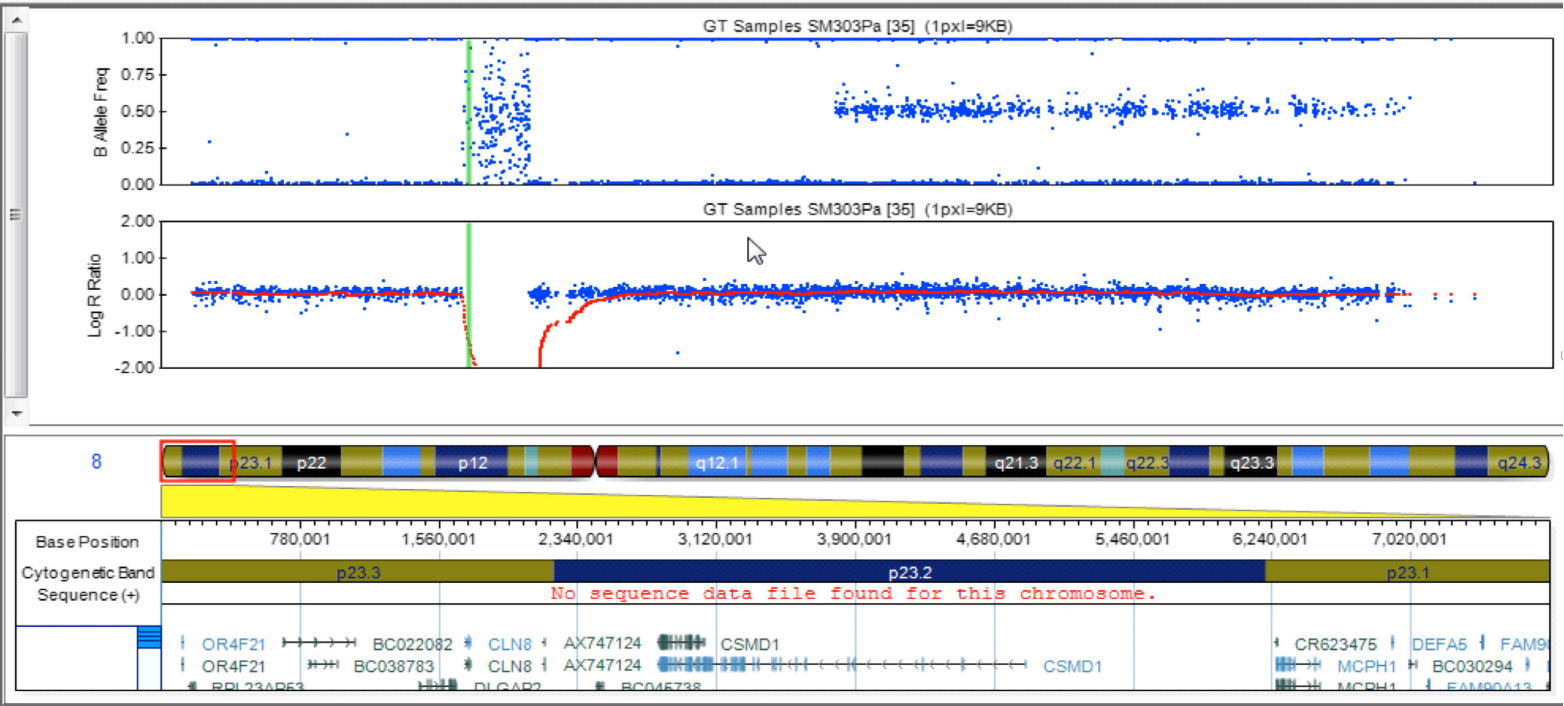
Results for chromosome 8 from whole genome genotyping represented by the log ratio in the bottom and B allele frequencies on the top panels. The green vertical bar indicates the location of CLN8 in chromosome 8. A large homozygous deletion on 8p23.3 encompassing CLN8 can be identified in Child A by the abrupt decline in the log ratio accompanied by randomly placed markers in the B allele frequency showing no specific hybridization with any probe in the array (Reference sequence: NM_001042432.1).

Whole genome genotyping revealed a large homozygous deletion of 378.6 kb on 8p23 that encompassed the *CLN8* gene as well as three other genes (Fig. 1).

### Child B

The proband from this British family had normal development with some mild language delay until the age of 4 years. From then onwards, her parents became concerned about worsening gait unsteadiness, loss of ability to climb stairs, and increasing difficulties with fine motor tasks. She experienced her first seizures at 4 years, 6 months, characterized by sudden falls lasting for seconds. On review at 4 years 8 months, she was showing myoclonic seizures, an ataxic gait, loss of fine motor skills, coordination difficulties, and visual impairment. The disease has been rapidly progressive with loss of independent ambulation and expressive language skills, and development of dysphagia requiring feeding support within 1 year of diagnosis. At 8 years of age, she was fully dependent on carers for all her needs. She died at 9 years and 2 months old.

Microscopy of a blood film showed no vacuolated lymphocytes. Electron microscopy of lymphocytes showed occasional lymphocytes containing membrane‐bound, electron‐dense, lipo‐protein storage material with a fingerprint profile (FP) suggestive of a variant form of NCL.

**Figure 2:**
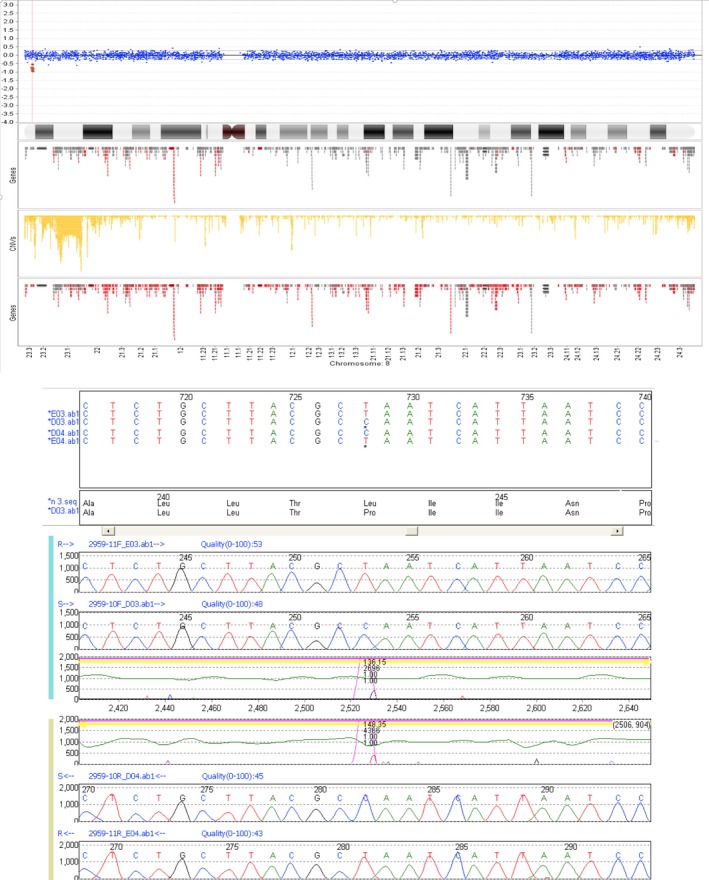
The upper panel is the array CGH result showing the heterozygous copy number loss of ~54 kb at 8p.23.3, which encompasses the *CLN8* gene (circled in red). The lower panel shows the Sanger sequencing results for the “hemizygous” c.728T>C, p.(Leu243Pro) novel variant in Child C. Upper and lower panels are the normal sequence (forward & reverse) and the middle panels are the patient (forward and reverse) (Reference sequence: NM_001042432.1).

Array‐CGH analysis (resolution of ~0.2 Mb) revealed a copy number loss (~54 kb) at 8p23.3 that encompasses the *CLN8* gene (Fig. 2). This copy number loss was too small to be confirmed by fluorescent in situ hybridization (FISH), and quantitative PCR was not validated in our laboratory at the time of detection. In addition, a MLPA dosage kit was also not available at that time. Therefore, we were unable to confirm the copy number loss by another method. However, microarray analysis in the father confirmed that he is a carrier of the same copy number loss, so the fact that it was inherited by the proband indicated that it was highly unlikely to be a false positive. Sequence analysis of *CLN8* indicated that the proband was also “hemizygous” for a novel variant c.728T>C p.(Leu243Pro). Leucine and proline have a moderate physicochemical difference and the leucine at residue 243 is conserved in 6/8 species. The proline residue is predicted not to be tolerated at this position and the amino acid change is predicted to be possibly damaging by in silico software. It is therefore considered likely to be pathogenic. Analysis of parental samples confirmed that the mother is a carrier of the c.728T>C variant. Together, these data confirm that the proband is a compound heterozygote for the 54 kb deletion and the c.728T>C variant.

### Child C

This male child is from a nonconsanguineous British family. There were no developmental concerns until the onset of a progressive visual impairment from 3 years 8 months, and features consistent with a retinal dystrophy. At 4 years of age, he was neurologically normal aside from the severe visual impairment with no evidence of seizures or cognitive decline. Brain imaging showed white matter abnormalities and he was initially investigated for a progressive leukodystrophy. He developed possible seizures at the age of 4 years 9 months and was started on medication following the EEG finding of abnormally slow background activity and runs of epileptiform discharges. By 5 years and 2 months of age, he had a marked Parkinsonian movement disorder with difficulties initiating movements and a shuffling gait. Seizures were well controlled. Anti‐Parkinsonian treatment was considered but within 6 months, he had lost all independent ambulation, speech, play, and feeding skills. A gastrostomy was placed. By 7 years of age, he was completely dependent for all his needs. He has prolonged episodes of restless and distressing hyperkinetic limb movements which have only partially responded to medication.

**Figure 3:**
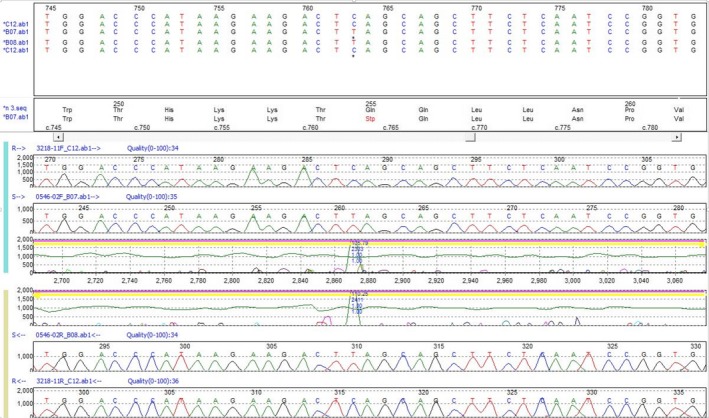
Sanger sequencing results for Child C showing the “hemizygous” c.763C>T, p.(Gln255*) mutation. Top and bottom panels are the normal sequence (forward and reverse) and the middle panels are the patient (forward and reverse) (Reference sequence: NM_001042432.1).

Microscopy of a blood film showed no vacuolated lymphocytes but electron microscopy showed approximately 8% of lymphocytes containing membrane‐bound, electron‐dense, lipo‐protein material with a fingerprint profile, suggestive of a variant form of NCL. All four genes associated with vNCL, *CLN5*,* CLN6*,* CLN7*/*MFSD8*, and *CLN8*, were Sanger sequenced and he was apparently homozygous for the c.763C>T, p.(Gln255*) nonsense mutation of *CLN8* (Fig. 3). However, parental studies showed that the father was a carrier of the c.763C>T mutation, but the mother was not. Subsequent microarray analysis showed that the proband was heterozygous for a 235 kb deletion in 8p23.3, which encompassed the *CLN8* gene, and was maternally inherited.

## Discussion

Our study of these families revealed three new genomic deletions that encompassed the *CLN8* gene. In one family, this was present on both disease alleles. In two families, their deletions unmasked two novel point mutations. The family with the largest deletion (Family A) had an interesting phenotype which may be due to the loss of additional nearby genes. Microscopically visible distal 8p deletions are known to be associated with growth and mental impairment, minor facial anomalies, congenital heart defects, and behavioral problems – these are features of Cornelia de Lange syndrome (OMIM **#**122470). However, although the clinical phenotypes of these cases show variability, this is within that previously described for late infantile onset CLN8 disease (Aiello et al. 2011) (Table 1).

**Table 1:** Comparison of families with large genomic deletions encompassing *CLN8*

Case	A (UCL303 Pa)	B	C	Allen et al. (2012)	Reinhardt et al. (2010) patients 3 and 4	Typical late infantile CLN8 disease
Country of origin	Argentina	UK	UK	Eire	Turkey	–
Consanguinity	No	No	No	No	Yes	N/A
Developmental delay	?	No	No	Yes	?	Yes
Microcephaly	?	No	No	Yes, 9th centile	?	Yes
Demanding behavior	?	No	No	Yes	?	?
Speech delay	?	Mild	No	?	4 years	Yes
Psychomotor regression	4 years	Yes	?	Yes		Yes
Disease onset	3 years	4 years	3 year 8 months	4 year	2.5 years	3.5–4 years
Onset unsteady gait	4 years	4 year 1 month	5 year 2 months			4 years
Onset seizures	3 year 8 months	4 year 4 months	4 year 9 months	4.5 year	3/3.5 years	4–6 years
Onset myoclonus	3 year 6 months	4 year 4 months	?		4 years	5 years
Onset drop attacks	4 years	4 year 4 months	?			
Chairbound	?	By 5 year 9 months	By 7 years	5.5 year	5 years	6 years
Visual failure	?	By 4 year 8 months	From 3 year 8 months	?	6 years	7–9 years
Immobilization	?	5 year 9 months	?		8 years	9 year‐early teens
Vacuolated lymphocytes	Not done	No	No	No		No
Ultrastructural EM	CL in skin biopsy	FP in lymphocytes	FP in lymphocytes	CL and FP in skin biopsy; FP in lymphocytes	CL, FP	CL, sometimes with FP or GROD
Electrophysiology EEG, ERG, VER, EMG, NCS	VEP abnormal at 5 years SEP abnormal at 5 years	ERG absent at 5 years VEP – early components of high amplitude at 5 years	EEG abnormal 4 year 9 months	EEG: slow background, complex partial; ERG absent; VER reduced EMG normal; NCS normal		VEP and ERG by age 7, extinguished ERG by 10
Brain imaging		Cerebellar atrophy, low signal change abnormality in thalami bilaterally		Hyperintensity of white matter plus Cerebellar atrophy	Cerebral and cerebellar atrophy	Cerebral and cerebellar atrophy
Genetics (novel in **bold**)	**400 kb del (homozygous)**	**54 kb deletion** (paternal); **c.728T>C** p.(Leu243Pro) (maternal)	**235 kb deletion** (maternal); **c.763C>T** p.(Gln255*) (paternal)	c.562_563delCT, p.(Leu188Valfs*58) (paternal); 8p23.3 terminal deletion, de novo	c.544‐2566_590del2613	N/A

?, Not reported or not known.

For patients with neurodevelopmental disorders and any form of intellectual disability, microarray analysis has superceded karyotype analysis as the first‐line test in the majority of diagnostic laboratories, due to its increased sensitivity in detecting copy number variants (CNVs). Although whole exome sequencing (WES) and targeted gene panels are becoming much more widespread, there are still currently significant limitations in detecting CNVs and trinucleotide repeats, due to a high level of biases and artifacts. However, as both sequencing technologies and bioinformatics improve, these limitations may become less of an issue.

This analysis contributes to establishing the prevalence of deletions at 8p23.3 and adds to the spectrum of *CLN8* mutations. We now recommend including microarray analysis in any patient suspected of NCL who is apparently homozygous for a mutation that is not present in one of the parents or when the family has no known consanguinity.

## Conflict of Interest

All authors declare that there is no conflict of interest.
